# Research on water meter reading recognition based on deep learning

**DOI:** 10.1038/s41598-022-17255-3

**Published:** 2022-07-27

**Authors:** Yue Liang, Yiqi Liao, Shaobo Li, Wenjuan Wu, Taorong Qiu, Weiping Zhang

**Affiliations:** School of Mathematics and Computer Sciences, Nanchang University, Nanchang, China

**Keywords:** Mathematics and computing, Computer science

## Abstract

At present, there are still many old-fashioned water meters in the society, and the water department needs to send staff to read the water meter after arriving at the scene with a handheld all-in-one machine. However, there are many problems in this manual meter reading method. First, a large number of meter reading work leads to low efficiency of the entire water department, consuming a lot of time and energy, and high labor costs; second, the water meters in natural scenes have problems such as serious dial contamination and other environmental factors that interfere with the meter reading staff, and the results of the meter reader cannot be verified later. In response to these problems, this paper studies a deep learning method for automatic detection and recognition of water meter readings. This paper first introduces the existing in-depth learning models, such as Faster R-CNN, SSD, and YOLOv3. Then two datasets are sorted out, one is the original water table picture dataset, and the other is a dataset cut out from the water meter image with the black bounding box showing the water meter readings. Then two plans are proposed, one is the original water table image dataset, and the other is a dataset cut out from the water meter image with the black bounding box showing the water meter readings. Finally, by comparing the three models from different angles, it is determined that YOLOv3 in the second solution has the best recognition effect, and the accuracy rate reaches 90.61%, which can greatly improve work efficiency, save labor costs, and assist auditors in reviewing the read water meter readings.

## Introduction

In the current society, traditional old-fashioned mechanical water meters are still used in places where development is relatively backward or in remote mountainous areas, requiring staff to read the meters on site. In the meter reading work, the management personnel of the business office is responsible for the arrangement of the meter-reading personnel, the adjustment of the meter-reading circuit and the formulation of the meter-reading plan. The meter reader downloads the meter reading task to the water supply management department through the spot by hand, and then carries the meter reading machine to the spot to read the meter. After completing the meter reading plan for the day, the meter reading result is uploaded to the meter reading system of the water supply management department. In this working mode, there are subjective factors such as deviation of observation angle or long distance when manually reading the numbers of the water meter, and problems such as dial contamination and limited energy of the meter reader, etc. It is difficult to arrange the meter reading plan, and it is impossible to judge whether the meter reading results reported by the meter readers are accurate (such as misreading or missing reading, etc.). Therefore, there are many problems in manual meter reading, and effective means are urgently needed to improve work efficiency and reduce labor costs.

In recent years, the development of artificial intelligence technology has become more and more mature, there are more and more researches on applying artificial intelligence technology to character recognition^1^. With the development of artificial intelligence technology becoming more and more mature, there are more and more researches on applying artificial intelligence technology to character recognition. The traditional digital recognition of water meter images usually consists of the following parts: one is to denoise the image to reduce the noise that interferes with the recognized numbers in the image, so that the numbers are clearer; the other is to binarize the image to separate the numbers in the image from the background, convert the image with three channels (RGB) into a grayscale image, and then convert it into a binary image with only 0 and 1^2^; the third is to segment the area where the character is located, and then perform tilt correction on the area to obtain a single character that is easy to identify Text image; Fourth, accurate recognition of a single character. However, the traditional solution is easily disturbed by the external environment, and the process is complicated. Different solutions need to be designed for different problems, and there is no way to accurately and quickly identify the collected real water meter images.

In recent years, deep learning algorithms have further developed and matured. Deep learning learns a large amount of data and automatically extracts features, and then trains specific algorithm models to obtain output results^3^. Deep learning is widely used in various aspects, and target detection is one of them. The role of target detection is to detect specific categories of targets on pictures, which can be widely used in various fields of production and life, such as military field^4^, biological field^5^, industrial manufacturing field^6^ and Object detection field^7^. With the maturity of image recognition technology in computer vision, more and more target detection algorithms have been proposed^8^. From R-CNN^9^ to MASK R-CNN^10^, R-CNN series (regions with CNN features), YOLO^11–13^ series (You only look once), SSD^14^ (Single Shot Multi Box Detector) and so on, the recognition accuracy in improving step by step, both the speed of recognition.

Compared with traditional identification methods, deep learning algorithms are less disturbed by external factors and have higher detection and identification accuracy^15^. Therefore, the Internet and deep learning technology are applied to meter reading companies to process water meter images and automatically detect and identify. The reading of the water meter becomes possible. Compared with the traditional recognition method, the water meter image recognition method based on deep learning can directly output the water meter numbers in the image in the form of a sequence, which not only has a fast detection speed, but also has a high recognition accuracy.

At present, deep learning methods in the field of object detection are mainly divided into two categories: Two-Stages and One-Stage. One-Stage refers to a target detection algorithm that directly converts the problem of target frame positioning into a regression problem without generating candidate frames. Common algorithms include YOLO, SSD, etc., so we choose classic SSD and YOLOv3 for experiments. In Two-Stages, it refers to a target detection algorithm that first generates a series of candidate frames as samples by an algorithm, and then classifies the samples through a convolutional neural network. Common algorithms include R-CNN, Fast R-CNN, Faster R-CNN^16^, etc., so we chose the classic Faster R-CNN algorithm for experiments.

In this paper, the target detection algorithm is used to quickly identify the water meter image taken by the meter reader and compare it with the manually read water meter number, screen out the wrong data and check it, which can help the auditor to reduce the workload, improve work efficiency, and then Reduce labor costs, help companies to better read water meters and increase revenue.

The structure of this article is as follows:

(i) *Introduction* This section introduces the basic process of meter reading in the water department, elicits the existing problems of manual meter reading, and introduces the research situation of traditional methods and deep learning methods in the field of water meter image recognition in recent years.
(ii) *Related studies* This section introduces the basic concepts of the three object detection algorithms used in this experiment, and compares the advantages and disadvantages of different algorithms.
(iii) *Materials and methods* This section introduces the sources and preprocessing methods of the water meter image dataset, and investigates the construction and selection of a water meter image reading model.
(iv) *Experimental comparison and analysis* This section introduces the specific experimental process and comparative analysis, and proposes improved methods.
(v) *Conclusion* This section summarizes the research content and experimental results, analyzes the advantages and disadvantages of the model, and proposes improvement methods.

## Related studies

### Faster RCNN algorithm for target detection based on candidate regions

In Faster R-CNN, a candidate region detection box is generated by introducing Region Proposal Networks (RPN network^16^), and Anchor Box is introduced to integrate feature extraction, candidate region recommendation, bounding box regression and classification into a network. And share the features extracted by the convolutional layer. The backbone network for Faster R-CNN is VGG16. The algorithm consists of two steps: first, the RPN network determines whether the candidate box is the target or not, and then the target type is determined by the multi-task loss objective function. The specific algorithm steps are as follows:

(i) The convolutional layer extracts feature maps from the input images for subsequent networks and RPN networks.
(ii) After the feature map is input into the RPN network, the candidate region is obtained, and the candidate box matrix and its score are output.
(iii) The feature map and candidate regions are pooled to output the proposal Feature Map through the Roi pooling layer, and then sent to the subsequent full-connection layer.
(iv) The full-connection layer conducts classification and border regression according to proposal Feature Map to get the position and score information of the final detection box.

### Target detection SSD method based on regression

The SSD model takes VGG16 as the main network structure, and changes the last two full connection layers of VGG16 into the convolution layer, and adds four convolution layers to construct the SSD network structure. SSD uses regression mode to quickly detect the category and location of objects. Also, feature maps of different scales are extracted based on regions to detect target regions. A total of 6 feature maps were extracted from the SSD model for detection, which presented an inverted pyramid structure from large to small.

### YOLOv3 algorithm for target detection based on regression

The YOLOv3 model is composed of two structures, one is the feature extraction layer (Darknet53)^11^ and the other is the YOLO detection layer. Darknet53 network consists of convolutional layer and residual unit. The convolutional layers are made up of 1 × 1 and 3 × 3 convolutional kernels, using batch normalization and LeakvReLU activation functions at each convolutional layer. YOLOv3 generates the position of the bounding box and category information of the entire image through the neural network in one step. The feature map of a specific size is obtained through the feature extraction network, and the image is divided into *S* × *S* networks. The grid is responsible for detecting the target and predicting *N* bounding boxes, including position information (*x*, *y*, *w*, *h*) and a confidence level. This format uses *P*_*r*_*(Object)* × *IOU* to determine the accuracy information of the subject and the bounding box. When *P*_*r*_*(Object)* equals 1, it means there is an Object, and when *P*_*r*_*(Object)* is 0, it means there is no Object. *IOU* is the value of the union over the intersection ratio of the predicted border and the real border, and the maximum value of *IOU* can be used to predict the object category. If there are *K* classes, YOLOv3 will output *S* × *S* × *(N* × *(4* + *1* + *K))*. *S* × *S* is the size of feature graph, *N* is the number of boundary boxes, *K* is the number of categories, 4 represents *(x, y, w, h)*, and 1 represents confidence.

### Comparison of algorithms

The Faster R-CNN series use the pyramid model to solve the problem of R-CNN clipping scale changes, classify the region of interest, improve the speed of candidate frame collection, and have better detection effects for small objects, but the detection speed cannot reach The effect of real-time detection.

The YOLO series lose part of the accuracy, and the image is detected end-to-end, and the detection speed is faster than the two-stage Faster R-CNN. The mAP is greatly improved by introducing the Anchor box system of R-CNN. The YOLO series don’t perform regional sampling, so it has better performance on global information, but poor performance on small-scale information.

SSD is a model that balances the advantages and disadvantages of YOLO series algorithms and Faster R-CNN. The Anchor box size of SSD is calculated, which is more scientific than the fixed anchor box of Faster R-CNN. Faster R-CNN has higher accuracy mAP and lower missed detection rate recall, but because it is a two-stage algorithm, the detection speed is relatively slow. The YOLO series of algorithms are on the contrary, with fast detection speed, but relatively low accuracy and missed detection rate.

## Materials and methods

**Figure 1:**
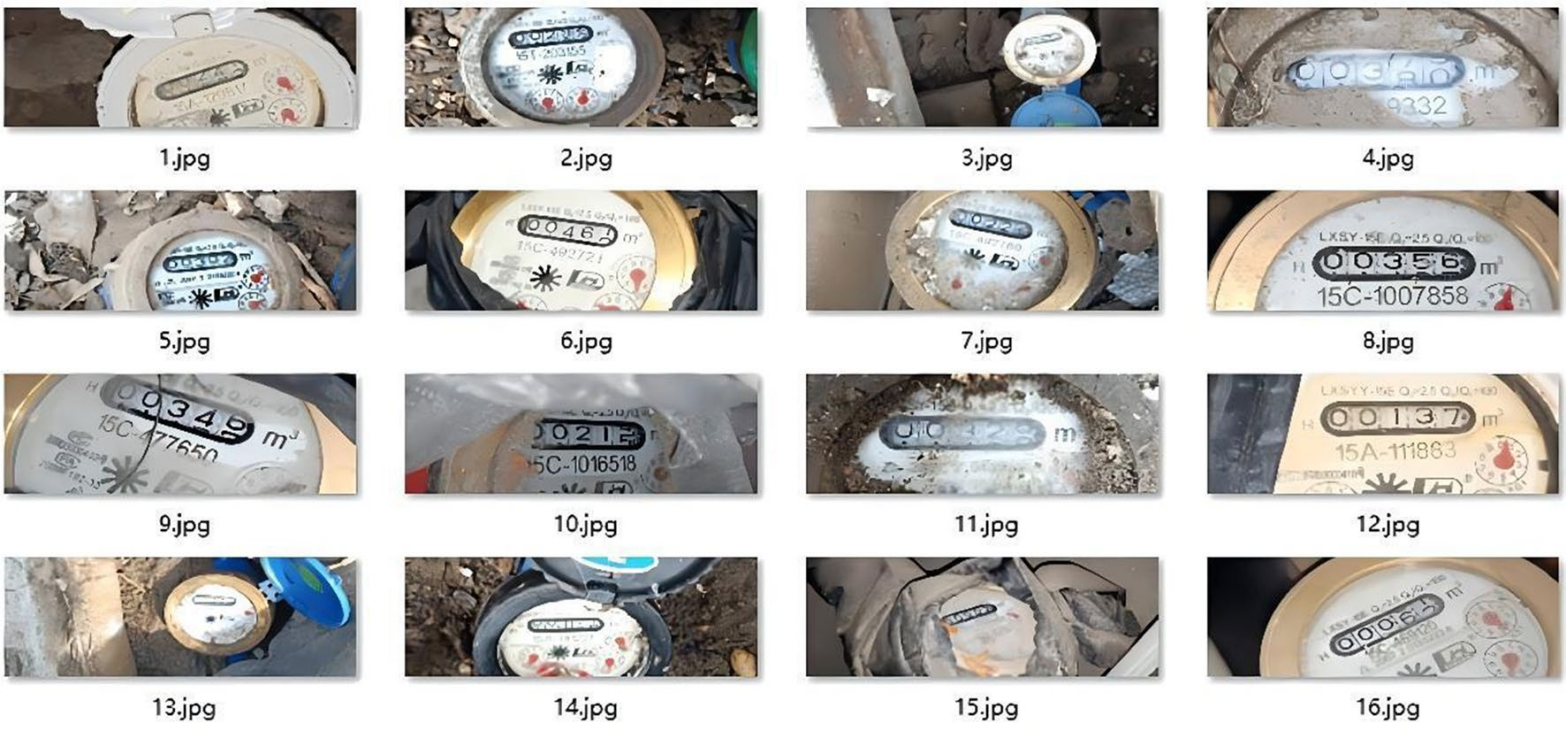
Part of water meter image display.

This data is obtained by meter reading personnel from 5 water supply branches and subordinate offices of Jiaxing Water Supply and Drainage Co., Ltd., who take pictures of water meters in real scenes with mobile phone cameras within the specified scope. Some images of water meters are shown in Fig. 1.

### Preprocessing of water meter image data set

#### Build a black bounding box for target detection network recognition

Due to the obvious features of the black bounding box, it is easy to identify. After comparative analysis of Faster R-CNN, SSD and YOLOv3, this paper selects the YOLOv3 target detection network with good recognition accuracy and speed to identify and cut the black bounding box, and obtains the black bounding box water meter image data set. The steps to build a YOLOv3 network are as follows.

**Figure 2:**
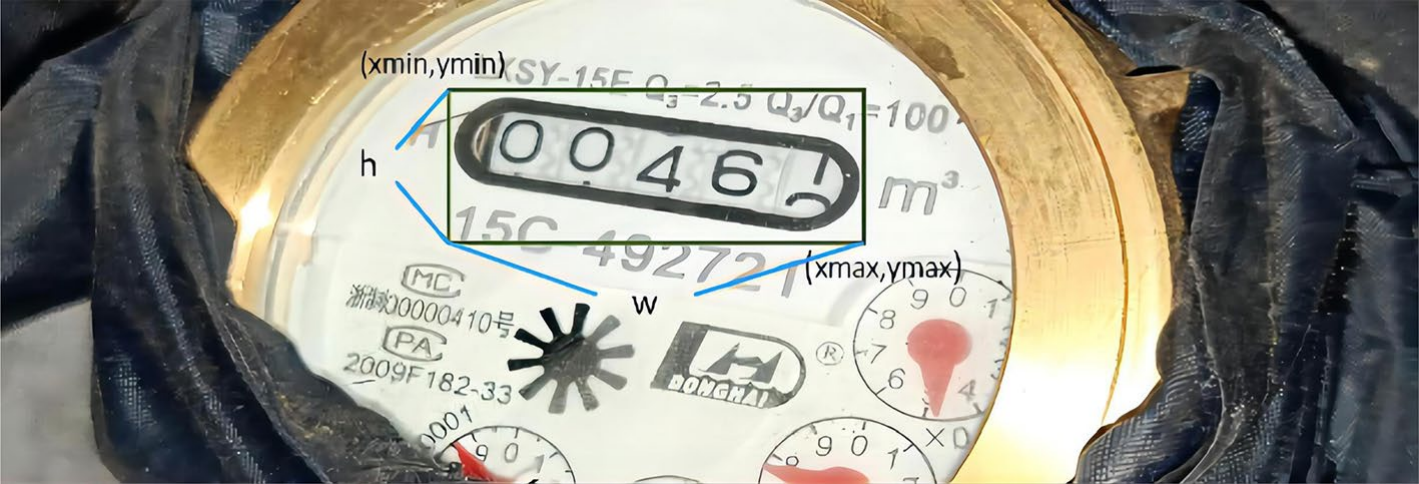
Picture label effect drawing.

First, Calculate the width and height of the mark. All the black bounding boxes are manually marked as the same class(this class is named screen) through the labelImg widget, generate xml files one by one, read out the coordinates in the xml file, calculate the width and height of the mark box, namely *w* and *h*, by calculating the values of *xmax − xmin* and *ymax − ymin*. The image labeling effect is shown in Fig. 2.

Second, Clustering algorithm is used to design the target detection box which conforms to the standard. Calculate all *w* and *h* of the annotation box and gather 9 center points to design 9 target detection boxes.

**Figure 3:**
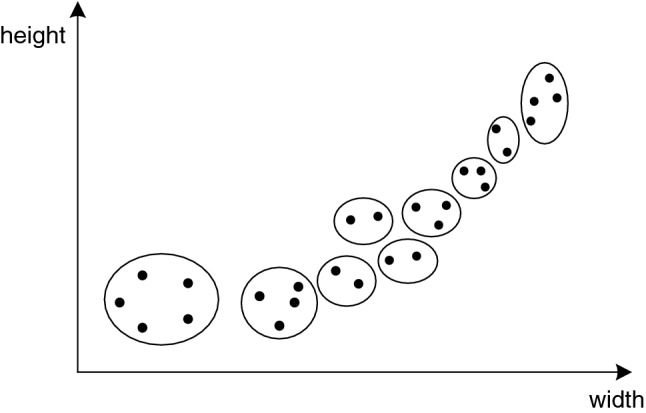
K-means classification effect diagram.

Nine target detection boxes are gathered by K-means algorithm. Build an array object with each group w and h. There are 3600 array objects in total, form a two-dimensional array, initialize 9 data points as the center of mass (*w*_1_, *h*_1_), (*w*_2_, *h*_2_), (*w*_3_, *h*_3_), …, (*w*_9_, *h*_9_), that is, nine classes. Calculate the distance from the center of mass for each array object in a two-dimensional array, such as *(w*_*a*_, *w*_*b*_*)*. If *(w*_*a*_, *w*_*b*_*)* is closer to (*w*_1_, *h*_1_), then (*w*_*a*_*, w*_*b*_) is divided into the class (*w*_1_, *h*_1_). After the first cycle, for each class, the average value of w and the average value of h are calculated to obtain *(w*_1_*′*, *h*_1_*′)* as the new center of mass, and then the distance and new center of mass are recalculated until no new center of mass position is generated. The classification effect Fig. 3 is as follows.

Hierarchical clustering method was used to divide 9 target detection boxes. 3600 array objects equals 3600 clusters. The Euclidean distance between clusters is calculated to determine whether it is the same class. Define a distance threshold. If the distance between clusters is closer and does not exceed the threshold (the threshold is set as 130 here), the two clusters are classified into one cluster; if the threshold is exceeded, the two clusters are divided into two categories. The center of mass of the clusters is represented by the mean value of the pair-distance of their respective data points.

**Figure 4:**
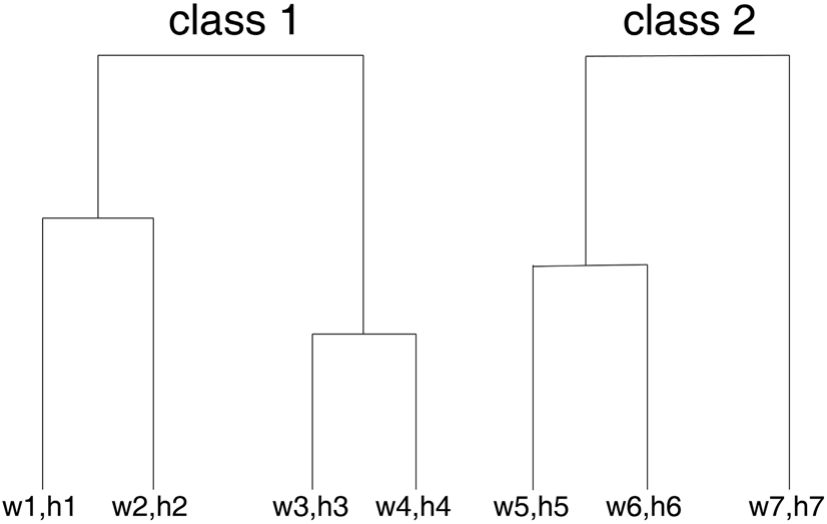
Partial renderings of hierarchical clustering.

Part of the effect of hierarchical clustering is shown in Fig. 4.

The classification comparison between K-means and hierarchical clustering algorithm is shown in Table 1.

**Table 1:** Comparison of clustering algorithms.

Clustering algorithm	Total width difference	Total height difference	Average width	Average height
K-means	593.23	113.39	210.02	60.42
Hierarchical clustering	67.58	14.48	310.51	84.98

**Figure 5:**
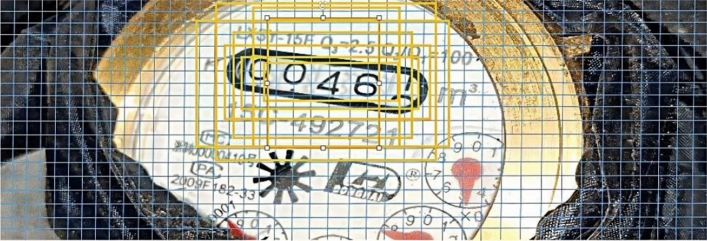
Effect of water meter image target box.

It can be seen from the figure that, compared with the classes divided by hierarchical clustering, the categories divided by K-means are more obvious in width and height, and can better classify the target box of identifying large target, medium target and small target. Combined with the water meter image data set, K-means can be used for better classification. The effect of some image target boxes is shown in Fig. 5.

**Figure 6:**
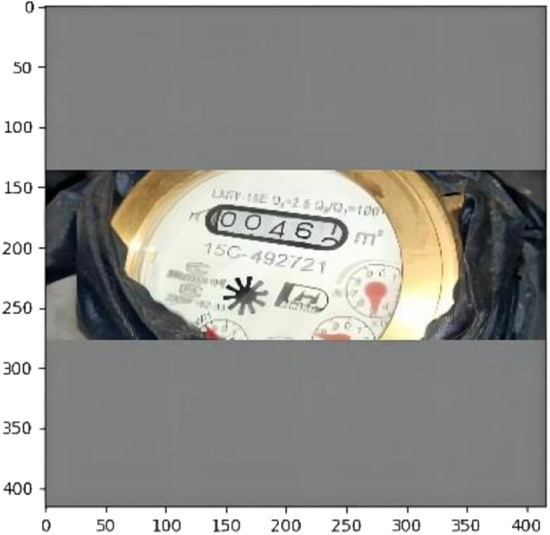
Image of water meter with uniform size.

Third, The training model of migration. In this experiment, model parameters pre-trained by COCO were selected for fine-tuning. The specific training steps are as follows:

- Preprocess the water meter image, unify the image size, for example: 416 × 416, shrink the long side to 416, shrink the short side and fill it with pixel RGB (128, 128, 128). The processed image is shown in Fig. 6.
- Select the pre-training model. In this experiment, model parameters pre-trained by COCO were selected for fine-tuning.
- Process the data set and set the data set to VOC format.
- Set the parameters of the experiment. Some parameter Settings are shown in Table 2. Epoch represents the number of training sessions, 30 of 1800 each. Batch size represents the two images used at each time. After repeated experiments, the batch size of 2 can meet the environmental requirements of this experiment. The learning rate is set between 1e−6 and 1e−4.
- Training and testing.

**Table 2:** Identifies part of the network parameters of the black bounding box.

Parameter names	Parameter values
BATCH_SIZE	2
IOU_LOSS_THRESH	0.5
ANCHOR_PER_SCALE	3
LEARN_RATE_INIT	1e−4
LEARN_RATE_END	1e−6
EPOCH	30 × 1800

#### The data of the black bounding box is processed

**Figure 7:**
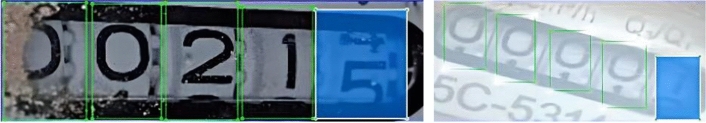
Annotations of some water meter images.

When marking the image, considering that there are numbers in other areas of the dial, in order to further eliminate the interference of other numbers, the black edge above and below the number of the water meter is also put into the box when marking. The effect is shown in Fig. 7.

**Figure 8:**
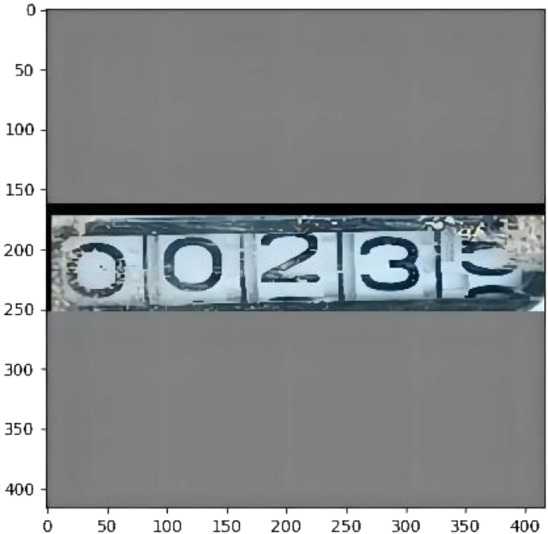
Water meter image after resizing.

Resize the image according to the network, such as 416 × 416, as shown in Fig. 8.

#### Split the training dataset and the test dataset

The initial water meter images in this paper are 4000 pieces, and the water meter images after the segmentation of the black rectangle are 4000 pieces in total. In this paper, the two data sets are randomly divided into 10 pieces, 9 pieces as training datasets and 1 piece as test data sets. Therefore, the two data sets respectively have 3600 pieces of training data sets and 400 pieces of test data sets.

#### Data enhancement of water meter image

Data enhancement is to expand more data by changing the picture data of the training and synchronously changing the position information of the label, but not changing the category of the label, so as to improve the quality of the picture. Meanwhile, this paper only expands the training set. In computer vision, many image transformation methods are used to expand data sets. In this paper, the following data expansion methods are used in the image preprocessing stage.

**Figure 9:**
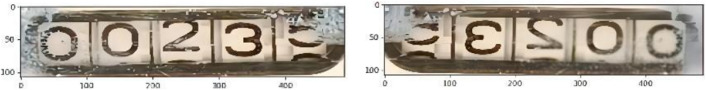
Water meter image after geometric transformation.

- The original image was flipped left and right to expand the data set and increase the diversity of the data.
- The original image is cropped randomly. Set the random function and the clipping threshold (for example, 0.5). If the value of the random function is less than the clipping threshold, it will be clipped randomly; otherwise, it will not be clipped randomly.
- Translate the original image. The fruit images in the data set were randomly shifted horizontally within the range of [0, width × 0.1] or vertically within the range of [0, height × 0.1].
- After the above geometric transformation, the images were amplified by about 3 times, and the whole training set was expanded to 14,400 images. An example diagram of a water meter turning left and right is shown in Fig. 9.

### Construction and selection of reading recognition model

#### Deep learning model building environment

Faster R-CNN uses the candidate boxes generated by RPN algorithm, and the candidate boxes are the same network as the CNN network for target detection, which reduces the number of candidate boxes to 300 and improves the quality of the selection boxes at the same time. To solve the problem of slow speed in Faster R-CNN, SSD adopts the idea of feature stratifying extraction, border regression and scoring simultaneously, and integrates the Anchors idea in Faster R-CNN, which is suitable for multi-scale target detection and faster at the same time. YOLOv3 is a target detection network with a very balanced speed and accuracy. Combined with this experimental environment, this paper uses Faster R-CNN in the two-stage target detection algorithm and SSD and YOLOv3 in the one-stage target detection algorithm to train the classification model of water meter graphics. The introduction of the experimental environment is shown in Table 3.

**Table 3:** Introduction to the experimental environment.

Parameter names	Parameter values
CPU	AMD Ryzen 7 1700 Eight-Core Processor 3.00 GHz
RAM	16 GB
Operating system	Windows64
GPU	NVIDA GeForce GTX 1060 6 GB
Programming language	Python3.7
Computer image vision library	OpenCV
Deep learning framework	TensorFlow2
CUDA vision	10.0

#### Selection of model plan

First, Faster R-CNN is constructed to recognize water meter numbers. The specific training steps are as follows:

- Make the data set in VOC format. There are 14,400 trainval files (training set) and 400 test files (test set).
- Debug the environment. The .pyx files of cython_bbox and bbox are compiled to generate .c files so that the files can be run on windows to generate bounding box.
- Determine the pre-training model. Select the public VGG16 as the pre-training model and fine-tune its parameters.
- Debugging parameters are shown in Table 4.
- Training and testing.

**Table 4:** Faster R-CNN partial parameters.

Parameter names	Parameter values
BATCH_SIZE	32
LEARNING_RATE	0.000001
EPOCH	12,000
RPN_POSITIVE_OVERLAP	0.7
RPN_NEGATIVE_OVERLAP	0.3

Second, construct SSD to identify water meter image. The specific training steps are as follows:

- Preprocessing on the water meter image. Scale the size of the image to 300 × 300. Make VOC data sets and convert images and labels to TF Records format.
- Determine the pre-training model and fine-tune it using the public VGG16 model parameters.
- Set experimental parameters as shown in Table 5. Since the image size is changed to 300 × 300, the batch size setting of 3 can best meet the requirements of the experiment. When the learning rate is greater than 0.001, the loss function of the model does not converge. Through many experiments, 1e−6 was selected as the learning rate of this experiment.
- Training and testing.

**Table 5:** Partial experimental parameters of SSD.

Parameter names	Parameter values
BATCH_SIZE	3
END_LEARNING_RATE	1e−6
EPOCH	12,000
MATCH_THRESHOLD	0.5
NUM_CLASS	11
TRAIN_IMAGE_SIZE	300

Third, construction of YOLOv3 recognition water meter image. The specific training steps are as follows.

Nine Anchorbox were selected by clustering algorithm. By calculating *w* and *h* of each annotation box, an array object is constructed with each group of *w* and *h*. There are a total of 16,402 array objects. The K-means algorithm and hierarchical clustering algorithm described in the second step of “Deep learning model building environment” section are used respectively to gather 9 AnchorBox classes. The experimental results are shown in Table 6.

**Table 6:** Experimental results of K-means and hierarchical clustering.

Clustering algorithm	Total width difference	Total height difference	Average width	Average height
K-means	93.8	106.5	30.62	34.86
Hierarchical clustering	13.88	16.56	40.54	47.84

It can be seen from the experimental results that the 9 AnchorBox classes gathered by the K-means clustering algorithm are more average compared with the hierarchical clustering algorithm, and can be used for multi-scale recognition with sizes ranging from [20, 23] to [114.03, 129.53], which is more in line with the design of YOLOv3. So select the 9 AnchorBox separated by K-means.

Use transfer learning. The specific training steps are as follows:

(i) Resize the image to 416 × 416.
(ii) Pre-training model selection. In this experiment, model parameters pre-trained by COCO were selected for fine-tuning.
(iii) Processing data sets. Put the data in VOC format and generate voc_train and voc_test texts.
(iv) Set experimental parameters. Parameter Settings are shown in Table 7.
(v) Training and testing.

**Table 7:** Some parameters of YOLOv3.

Parameter names	Parameter values
BATCH_SIZE	2
IOU_LOSS_THRESH	0.5
ANCHOR_PER_SCALE	3
LEARN_RATE_INIT	1e−4
LEARN_RATE_END	1e−6
EPOCH	30 × 7200

## Experimental comparison and analysis

### Program design and improvement

In this paper, there are two plans for the outline design of water meter digital recognition model.

**Figure 10:**
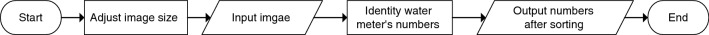
Flow chart of Plan A.

Plan A: Overall picture recognition. Image preprocessing stage, the image processing into a uniform format size, and the use of transfer learning. In the training stage, the water meter pictures were input into the deep learning model to learn ten categories. Categorize each goal. According to the predicted result, sort by the position size of x. Finally output the number on the water meter. The main process is shown in Fig. 10.

**Figure 11:**
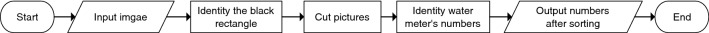
Flow chart of Plan B.

Although the Plan A can identify the number in one step, the target will detect other numbers on the dial due to the existence of some external factors such as illumination, dust, interferences and some digital interference of the dial itself. Therefore, Plan A is improved on this basis and Plan B is proposed, as shown in Fig. 11.

Plan B: The image is recognized step by step. The first step of image preprocessing, adjust the picture size, and then carried out on the water meter image target detection for the first time, select YOLOv3 target detection depth learning network, and then will deal with good image input to the network, and learn a category, namely, with black rectangle category (screen), and used to predict the *(x, y, w, h)* to cutting of pictures to process the dataset further. The second step is to retrain the model and learn from 10 categories (0–9). The two-character criteria are used to determine the number based on the larger proportion of numbers in a box. According to the predicted results, the x position is sorted by the predicted size. Finally output the number on the water meter.

### Comparison of model and plans results

mAP (mean average precision) is a commonly used evaluation index in multi-task target detection. Firstly, whether the prediction box or the prediction box is TP or FP is determined according to whether the IOU of the prediction box and the real box of each figure is greater than 0.5. Then, the prediction box is ranked from high to low according to the confidence of each prediction box, and the Precision and Recall under different confidence threshold values are obtained. PR curve is drawn and the area is obtained. AP is an indicator of the detection quality of a class, and mAP is an indicator of the detection quality of multiple classes, namely the average value of multiple categories of AP. Since the model needs to be used in the project, there are certain requirements for the accuracy. The higher the accuracy, the more efficient the user will be. In addition, for the prediction speed and model size of the model, the better the better on the basis of not affecting the accuracy requirements. To sum up, the evaluation indexes of the model are mAP, accuracy, recognition speed and model size.

First, Experimental results of identifying black bounding boxes are shown in Table 8.

**Table 8:** Experimental results of identifying black bounding boxes.

Models	mAP (%)	Recognition speed (ms/piece)	Model size (MB)
Faster R-CNN	87.48	382.74	108.1
SSD	89.26	**60.15**	**95.84**
YOLOv3	**91.30**	66.87	231.1

The bold values are meant to represent the best results obtained under the corresponding metric.

**Figure 12:**
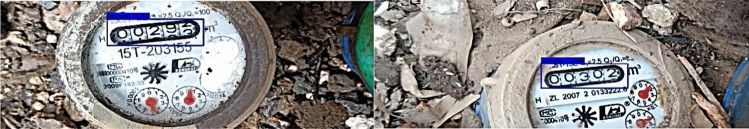
Display of detection images of some water meters with black bounding boxes.

It can be concluded that YOLOv3 has a significant effect on the recognition of the black bounding box, can accurately identify the target area, and eliminate the interference of lighting, stains and black background. On this basis, this paper cuts the predicted target region and makes a data set. Some images are shown in Fig. 12.

**Figure 13:**
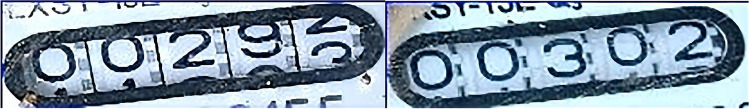
Shows a picture with a black bounding box cut out.

The image is cut according to the predicted *(x, y, w, h)*, and the cut part of the image is shown in Fig. 13.

At this point, this paper obtains a cut data set. According to the regulations of the company, this paper divides the numbers of water meters into ten categories. For single characters, each number represents a category; for double characters, the number with a large proportion represents the whole category of double characters.

Second, The result of identifying the water meter picture after cutting.

**Figure 14:**
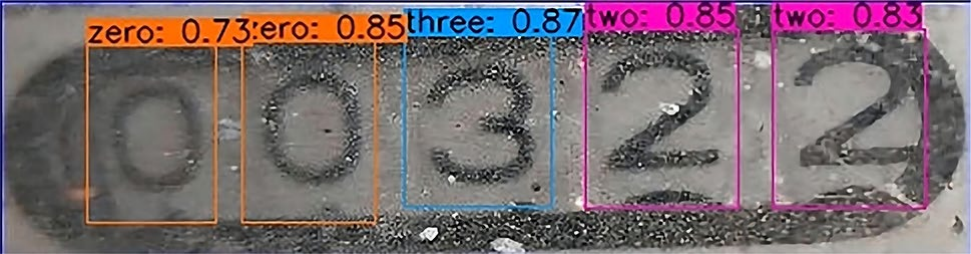
A partial rendering of the Faster RCNN test.

**Figure 15:**
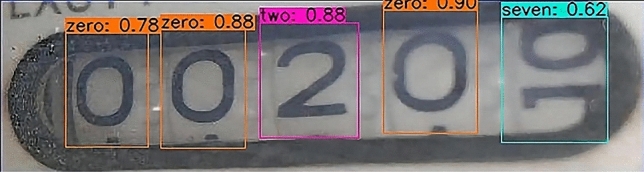
Partial renderings of SSD tests.

**Figure 16:**
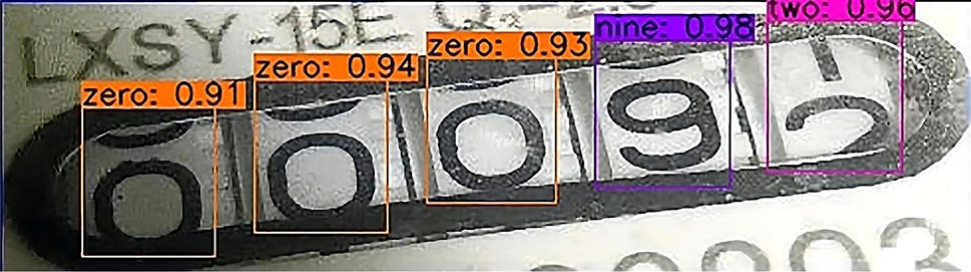
A partial rendering of the YOLOv3 test.

- A partial rendering of the Faster R-CNN test is shown in Fig. 14.
- The experimental results of SSD are shown in Fig. 15.
- The results of YOLOv3 experiment are shown in Fig. 16.

Third, Comparison of experimental results of the Plans. The experimental results of Plan A are shown in Table 9, and The experimental results of Plan B are shown in Table 10.

**Table 9:** Experimental results of Plan A.

Models	mAP (%)	Accuracy	Recognition speed (ms/piece)	Model size (MB)
Faster R-CNN	77.07	82.96	397.6	111.5
SSD	75.67	78.63	**61.62**	**98.27**
YOLOv3	**78.10**	**87.30**	68.54	234.8

The bold values are meant to represent the best results obtained under the corresponding metric.

**Table 10:** Experimental results of Plan B.

Models	mAP (%)	Accuracy	Recognition speed (ms/piece)	Model size (MB)
Faster R-CNN	75.46	81.53	395.1	109.4
SSD	78.61	80.42	**60.42**	**98.58**
YOLOv3	**82.02**	**90.61**	69.84	232.5

The bold values are meant to represent the best results obtained under the corresponding metric.

AP values for individual characters in Plan B are compared as shown in Table 11.

**Table 11:** Experimental results of Plan A.

Singel character AP	Faster R-CNN	SSD	YOLOv3
Zero	79.85	83.11	**84.29**
One	74.64	77.46	**78.97**
Two	78.42	81.31	**83.77**
Three	80.31	82.99	**86.70**
Four	76.23	80.24	**82.94**
Five	79.59	81.87	**85.33**
Six	70.44	75.22	**80.45**
Seven	69.54	72.87	**81.33**
Eight	69.34	72.66	**77.11**
Nine	76.28	78.34	**79.36**

The bold values are meant to represent the best results obtained under the corresponding metric.

In combination with the results in the table it can be seen that for the water meter image data in the recognition, the best is YOLOv3 network, can accurately identify the water meter black bounding box in the digital images, and basic would not identify the black rectangle beyond the Numbers, if there is a character by two box predicted that if the two boxes of IOU value is greater than 0.8, By comparing the confidence, the greater one was selected as the predicted value, and the accuracy reached 90.61%. Compared with YOLOv3, the accuracy of SSD network is not very good at identifying the numbers in the black rectangle, and the proportion of SSD network identifying the numbers outside the black rectangle is higher than that of YOLOv3. The influence later is sorted according to the size of the predicted x. So it’s not as effective as YOLOv3. The accuracy of Faster R-CNN network is also not as high as that of Yolov3, and compared with YOLOv3, Faster R-CNN cannot effectively identify every data in the black bounding box. In addition, Faster R-CNN does not carry out good non-maximum suppression for the selection of candidate box, which leads to the recognition of multiple numbers into one number and the failure of candidate box to match the number region well, which does not meet the practical requirements.

**Figure 17:**
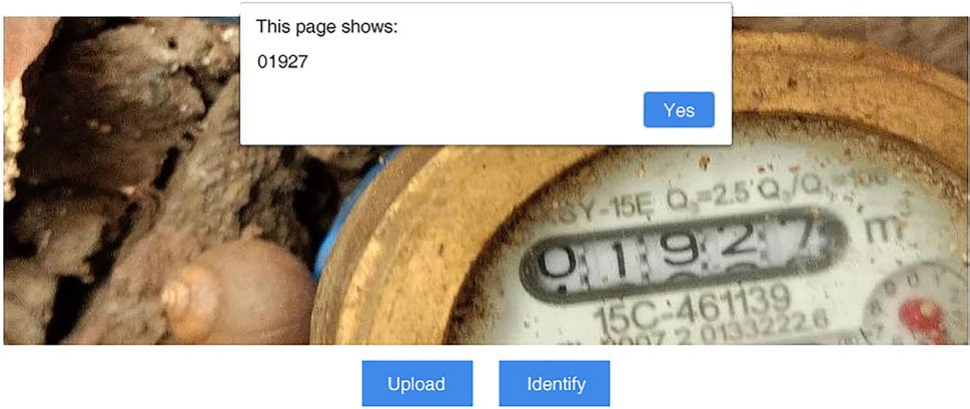
Test result diagram.

Fourth, Testing. Deploy the model to the server, and through the simple test on the page, the water meter number can be output effectively, as shown in Fig. 17.

## Conclusion

According to the actual demand of water meter reading recognition, this paper deeply studies and analyzes the existing deep learning model, and proposes two Plans. Plan B compared to Plan A, mAP of YOLOv3 improved by nearly 4 percentage points, and its accuracy rate increased by about 3 percentage points, reaching 90.61%. It can accurately identify the numbers in the black bounding box and output the sorted numbers, which can greatly improve labor efficiency and save labor costs.

In the following research work, we will learn and use the updated model to conduct research and experiments.

## Data Availability

The data that support the findings of this study are available from the offices of Jiaxing Water Supply and Drainage Co but restrictions apply to the availability of these data, which were used under license for the current study, and so are not publicly available. Data are however available from the corresponding author on reasonable request and with permission of the offices of Jiaxing Water Supply and Drainage Co.

## References

[1] Chen C (2021). Data dissemination for industry 4.0 applications in internet of vehicles based on short-term traffic prediction. ACM Trans. Internet Technol..

[2] Wang H (2021). Rib segmentation algorithm for X-ray image based on unpaired sample augmentation and multi-scale network. Neural Comput. Appl..

[3] Xu D, Wang L, Li F (2021). Review of typical object detection algorithms for deep learning. Comput. Eng. Appl..

[4] Zhang B, Jia J, Wang W (2021). Improvement of military target detection algorithm based on yolov3. Netw. Secur. Technol. Appl..

[5] Zhang M (2021). Method for moving object detection of underwater fish using dynamic video sequence. J. Graph..

[6] Zhuang, F. Comparative study and application of three target detection algorithms in recognition of welding spot position of automobile door panel. Ph.D. thesis, South China University of Technology (2021).

[7] Zhang L (2021). Vehicle object detection method based on candidate region aggregation. Pattern Anal. Appl..

[8] Wu Y, Ma Y, Wan S (2021). Multi-scale relation reasoning for multi-modal visual question answering. Signal Processing: Image Commun..

[9] Girshick, R., Donahue, J., Darrell, T. & Malik, J. Rich feature hierarchies for accurate object detection and semantic segmentation. In *Proc. IEEE Conference on Computer Vision and Pattern Recognition*, 580–587 (2014).

[10] He, K., Gkioxari, G., Dollár, P. & Girshick, R. Mask r-cnn. In *Proc. IEEE International Conference on Computer Vision*, 2980–2988 (2017).

[11] Redmon, J., Divvala, S. & Girshick, R. Yolo9000: Better, faster, stronger. In *Proc. IEEE Conference on Computer Vision and Pattern Recognition*, 6517–6525 (2017).

[12] Redmon, J. & Farhadi, A. You only look once: unified, real-time object detection. In *Proc. IEEE Conference on Computer Vision and Pattern Recognition*, 779–788 (2016).

[13] Redmon, J. & Farhadi, A. Yolov3: An incremental improvement. Preprint at http://arxiv.org/abs/1804.02767 (2018).

[14] Liu, W. *et al*. Ssd: Single shot multibox detector. In *Proc. European Conference on Computer Vision*, 27–31 (2016).

[15] Panagiotis R (2021). Modelling, detecting and mitigating threats against industrial healthcare systems: A combined SDN and reinforcement learning approach. IEEE Trans. Ind. Inform..

[16] Ren S, He K, Girshick R, Sun J (2017). Faster r-cnn: Towards real-time object detection with region proposal networks. IEEE Trans. Pattern Anal. Mach. Intell..

